# How social, psychological, and spiritual needs are supported in the last months of life: evidence from the English Longitudinal Study of Ageing end-of-life interviews

**DOI:** 10.1093/ageing/afag202

**Published:** 2026-07-06

**Authors:** Clément Meier, Nicola White, Andrew Steptoe, Libby Sallnow

**Affiliations:** Faculty of Business and Economics (HEC), University of Lausanne, Lausanne, Switzerland; Department of Epidemiology and Public Health, Institute of Epidemiology and Health Care, University College London, London, UK; Marie Curie Palliative Care Research Department, University College London, London, UK; Department of Behavioural Science and Health, Institute of Epidemiology and Health Care, University College London, UK; Marie Curie Palliative Care Research Department, University College London, London, UK; End of Life Care Research Group, Vrije Universiteit Brussel, Brussels, Belgium

**Keywords:** end-of-life care, palliative care, non-medical needs, psychological support, social support, spiritual support, population-based study, older people

## Abstract

**Background:**

End-of-life care includes social, psychological, and spiritual support in addition to medical care. Population-level evidence on whether these non-medical needs are met in the last months of life, who provides such support, and how it is perceived remains limited.

**Methods:**

We analysed end-of-life proxy interviews from deceased participants aged 55 years and older in the English Longitudinal Study of Ageing (*n* = 507). Outcomes included whether social, psychological, and spiritual needs were met in the last three months of life, and how support was provided and perceived. Analyses included multivariate probit models adjusted for sociodemographic and end-of-life factors.

**Findings:**

Individuals died at a mean age of 83 years (range 55–105). Social and psychological needs were met for most individuals (81.8% and 77.8%); among those with spiritual needs (*n* = 227), 74.4% had these needs met. Support was provided predominantly by family members, with limited involvement of formal services, and was generally rated as good or excellent and helpful. Living alone was associated with lower probabilities of having social, psychological, and spiritual needs met, while dementia was associated with lower probabilities of having social and psychological needs met (*P* < .05–.01). Higher educational attainment was associated with a higher probability of having spiritual needs met (*P* < .05–.01).

**Conclusions:**

Reliance on informal networks creates inequities in non-medical end-of-life support, particularly for older adults living alone or with dementia, underscoring the need for more integrated public health approaches.

## Key Points

- Non-medical end-of-life support relies predominantly on informal networks, especially family members.
- Older adults living alone are at increased risk of unmet social, psychological, and spiritual needs.
- Dementia is associated with higher likelihood of unmet social and psychological needs at the end of life.
- Strengthening integration between geriatric, community, and palliative care services is essential to reduce inequalities.

## Introduction

A global reduction in premature death and increasing life expectancy has led to a growing number of deaths occurring at older ages [1]. While life expectancy has increased, healthy life expectancy has not kept pace, meaning the last years of life can be marked by chronic illness, multimorbidity, and functional decline [2]. Serious health-related suffering describes suffering that requires healthcare intervention [3]. Palliative and end-of-life care responds to this suffering through attention to physical, psychological, social, and spiritual domains [4, 5]. However, access to palliative care remains inequitable [6], with the World Health Organization estimating that 14% of those in need globally are currently able to access it [4].

Though based on a holistic model of care, palliative and end-of-life care services have been criticised for a lack of holism in practice [7], prioritising physical symptoms and biomedical approaches at the expense of psychological, social, or spiritual issues [8]. Studies of social, psychological, and spiritual interventions by palliative care services have shown limited improvement in outcomes and often focused on single domains, specific care settings, or selected patient groups [9–11]. This limits insights into whether such needs are met, who provides support, and how support is perceived across different social contexts. Failure to meet these needs has been associated with increased emotional distress, poorer experiences of care, and unmet preferences at the end of life for both individuals and their families [12, 13]. Palliative care frameworks increasingly recognise that dying, caring, and grieving are complex processes, requiring support beyond clinical interventions [14]. A public health approach to palliative and end-of-life care emphasises the role family, friends, and wider community networks play in reliving suffering and building health and wellbeing at the end of life [15, 16]. It explicitly recognise the social and structural determinants of health in death, dying, and loss [17].

Recent studies highlight the contribution of informal carers in meeting social support needs and the role of participatory and public health interventions to support them [18, 19]. Family members, friends, and neighbours support older adults at the end of life, often in the context of constrained formal care resources and policies promoting ageing in place, increasing reliance on informal networks to meet non-medical care needs [20, 21]. Understanding the role of informal care networks, the quality of care received, the interface with formal health and social care services, and the sustainability of such networks in addressing social, psychological, and spiritual needs are critical to inform equitable palliative and end-of-life care policies and practice. At the same time, living alone, social isolation, and cognitive impairment are becoming more prevalent among older populations, raising concerns about unequal access to non-medical support at the end of life [22, 23]. Understanding which groups are most likely to experience unmet non-medical needs is therefore critical. This study examines whether social, psychological, and spiritual needs are met in the last three months of life, who provides this support, how it is perceived in terms of quality and helpfulness, and which sociodemographic and end-of-life characteristics are associated with unmet needs.

## Methods

### Study design and participants

This study analysed data from end-of-life proxy interviews collected as part of the English Longitudinal Study of Ageing (ELSA), a nationally representative study of adults aged 50 years and older living in England, designed to collect detailed information on health, socioeconomic circumstances, and social and family networks, running since 2002 [24]. End-of-life interviews are conducted with a close relative, friend, or carer of an ELSA core sample member who has died and collect information on the individual’s final year(s) of life, including health, care needs, social circumstances, and finances. The present study used 177 end-of-life interviews from ELSA’s Harmonised Cognitive Assessment Protocol (HCAP) round 2, administered between January and June 2023, and 347 end-of-life interviews from ELSA Wave 11, collected between March 2024 and January 2025. The analytical sample included deceased participants who died between 2016 and 2024, with ages at death ranging from 55 to 105 years. The interval between death and the end-of-life interview varied across respondents, as interviews are conducted during subsequent survey waves depending on fieldwork timing rather than at a fixed interval following death. As a result, the time elapsed between death and interview is not uniform but is typically within months to a few years. A total of 524 end-of-life interviews were initially available. Of these, 17 respondents were excluded due to missing information on variables used in the analyses. The final analytical sample therefore consisted of 507 deceased participants. ELSA received ethical approval through the Health Research Authority (22/SC//0325 and 23/SC/0112) and informed consent was obtained from all participants and proxy respondents.

### Outcome variables

The primary outcomes were whether the deceased’s social, psychological, and spiritual needs were met during the last three months of life. For each domain, respondents were asked whether the deceased’s needs had been met, with responses coded as binary indicators (yes or no). Spiritual needs were assessed only among individuals for whom the proxy respondent answered affirmatively to a screening question asking whether the deceased was religious or spiritual. For each domain in which needs were reported as met, additional questions captured the sources of support, perceived quality of support, and perceived helpfulness of support. Sources of support were measured using multiple-response items indicating whether different providers had helped to meet the deceased’s needs in the last three months of life. These included family members, friends, neighbours, healthcare services, social services, outpatient or community palliative care, voluntary organisations, religious organisations, and other sources. Perceived quality of support was assessed separately for social, psychological, and spiritual needs using a four-category scale (excellent, good, fair, or poor). Perceived helpfulness was measured by asking whether having these needs met helped the deceased cope with illness and being close to death, with responses coded as yes or no.

### Independent variables

Independent variables included a set of sociodemographic and end-of-life characteristics. Age at death was categorised into three groups (55–74, 75–84, and 85 years or older). Sex was coded as male or female. Living arrangement prior to death was captured as a binary indicator of whether the deceased lived alone. Educational attainment was classified into three categories (primary, secondary, and tertiary education). Socioeconomic position was measured using the most recent standardised non-pension household wealth score available prior to death, treated as a continuous variable, standardised to a mean of zero, with higher values indicating greater household wealth. Place of death was categorised as home, hospital, or other settings. Cause of death was grouped into cancer, cardiovascular-related illness, respiratory disease, and other causes. Finally, cognitive status at the end of life was captured using a binary indicator of whether the deceased had dementia or a severe cognitive condition.

### Statistical analysis

Descriptive analyses were used to summarise the characteristics of the study population and to describe the distribution of social, psychological, and spiritual needs met in the last three months of life, as well as sources, quality, and perceived helpfulness of support. Results are presented as frequencies and percentages. To examine associations between sociodemographic and end-of-life characteristics and having social, psychological, and spiritual needs met, multivariate probit regression models were estimated. Separate models were fitted for each outcome. Results are reported as average marginal effects (AME) with corresponding standard errors, allowing for interpretation as absolute differences in predicted probabilities. Models were adjusted for age at death, sex, living alone, educational attainment, standardised non-pension wealth, place of death, cause of death, and dementia or severe cognitive condition. All analyses were conducted using STATA/SE 18.0 (StataCorp, College Station, TX, USA). Statistical significance was assessed at the 5% level using two-sided tests.

## Results

All end-of-life interviews were completed by relatives or close contacts of the deceased, most commonly adult children (45.8%) and spouses or partners (37.5%). Table 1 summarises the characteristics of the study population (*n* = 507).

**Table 1:** Characteristics of the study population, ELSA end-of-life interviews, *n* = 507.

	*n*	%
**Age at death**		
55–74	96	18.9
75–84	182	35.9
85+	229	45.2
**Sex**		
Male	262	51.7
Female	245	48.3
**Living alone**		
No	291	57.4
Yes	216	42.6
**Education level**		
Primary	179	35.3
Secondary	251	49.5
Tertiary	77	15.2
**Standardised non-pension wealth**	mean: −0.49 min: −0.86	std. dev: 0.88 max: 11.12
**Place of death**		
Home	173	34.1
Hospital	202	39.8
Other	132	26.1
**Cause of death**		
Cancer	163	32.1
Cardiovascular related illness	104	20.5
Respiratory disease	81	16.0
Other	159	31.4
**Dementia or severe cognitive condition**		
No	382	75.3
Yes	125	24.7

*n*, number of observations for the whole sample.

**Figure 1:**
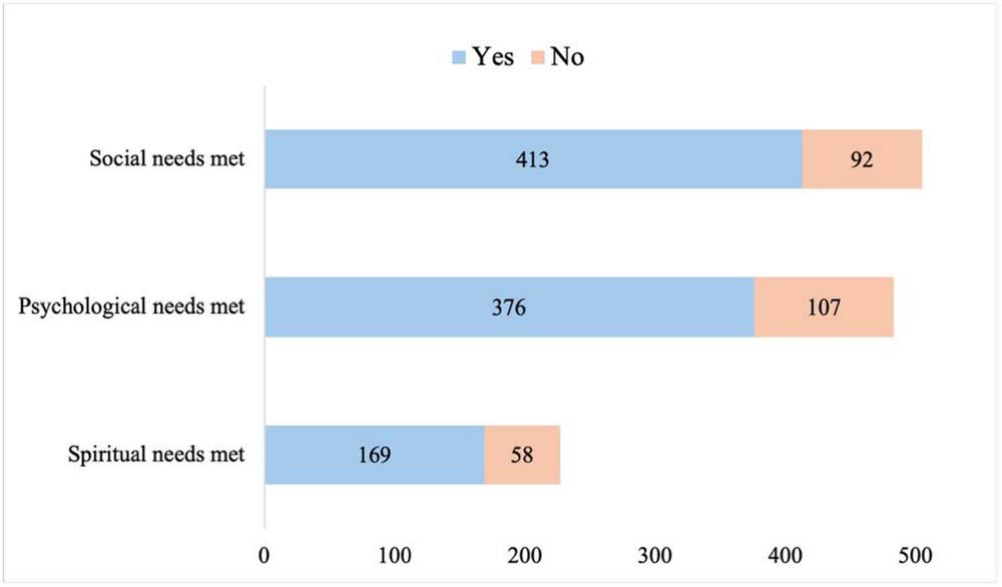
Distribution of social, psychological, and spiritual needs met in the last 3 months of life, ELSA end-of-life interviews.

The proportion of individuals whose social, psychological, and spiritual needs were met in the last three months of life are presented in Figure 1. Social needs were reported as met for 413 of 505 individuals (81.8%), while 92 (18.2%) reported that these needs were not met. Psychological needs were met for 376 of 483 individuals (77.8%), with 107 (22.2%) reporting unmet needs. Spiritual needs were relevant for a smaller subgroup; among those for whom these needs were reported as applying, 169 of 227 individuals (74.4%) reported that spiritual needs were met, while 58 (25.6%) reported unmet spiritual needs.

**Figure 2:**
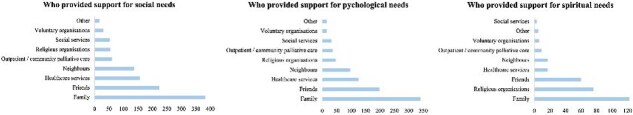
Distribution of who helped meet social, psychological, and spiritual needs in the last 3 months of life, ELSA end-of-life interviews.

Figure 2 shows the distribution of providers who helped to meet social, psychological, and spiritual needs in the last three months of life. Across all three domains, family members were the most frequently reported source of support. Friends and neighbours also contributed to support, particularly for social and psychological needs. Healthcare services were reported as a source of support mainly for social and psychological needs, while their involvement in spiritual support was less frequent. Outpatient or community palliative care, social services, voluntary organisations, and religious organisations were reported by a smaller proportion of respondents across domains, with religious organisations contributing primarily to spiritual support.

**Figure 3:**
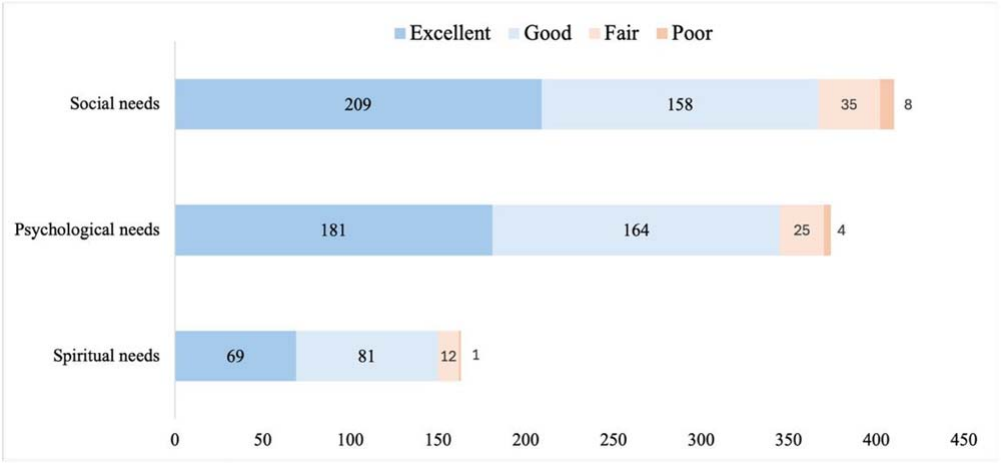
Distribution of the perceived quality of support for social, psychological, and spiritual needs in the last 3 months of life, ELSA end-of-life interviews.

The results from Figure 3 shows the perceived quality of support for social, psychological, and spiritual needs in the last three months of life among individuals whose informant indicated that they had received support. For social needs, support quality was most often rated as excellent (209 of 410; 51.0%) or good (158 of 410; 38.5%), while fewer respondents rated support as fair (35 of 410; 8.5%) or poor (8 of 410; 2.0%). A similar pattern was observed for psychological needs, with support rated as excellent by 181 of 374 individuals (48.4%) and good by 164 (43.9%), whereas 25 (6.7%) rated support as fair and 4 (1.1%) as poor. For spiritual needs, perceived quality was more evenly distributed between excellent (69 of 163; 42.3%) and good (81 of 163; 49.7%), with relatively few respondents reporting fair (12; 7.4%) or poor (1; 0.6%) quality.

**Figure 4:**
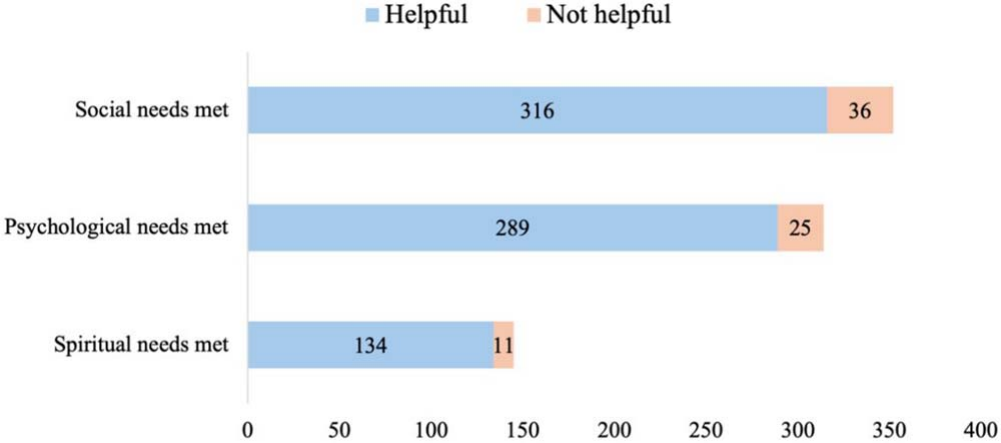
Distribution of perceived helpfulness of having social, psychological, and spiritual needs met in the last 3 months of life, ELSA end-of-life interviews.

Among individuals whose informant reported that they received support, most perceived having their social, psychological, and spiritual needs met as helpful in the last three months of life (Figure 4). Social support was perceived as helpful by 316 of 352 individuals (89.8%), while 36 (10.2%) reported that it was not helpful. Psychological support was perceived as helpful by 289 of 314 individuals (92.0%), with 25 (8.0%) reporting that it was not helpful. Similarly, spiritual support was perceived as helpful by 134 of 145 individuals (92.4%), while 11 (7.6%) reported that it was not helpful.

Table 2 presents adjusted associations between sociodemographic and end-of-life characteristics and having social, psychological, and spiritual needs met in the last three months of life. Living alone was associated with a lower probability of having social needs met (AME −0.08, *P* < .05), psychological needs met (AME −0.14, *P* < .01), and spiritual needs met (AME −0.15, *P* < .05). For example, an AME of −0.08 indicates an average 8 percentage-point lower probability of having social needs met compared with the reference group, after adjustment for other covariates. Having dementia or a severe cognitive condition was associated with a lower probability of having social needs met (AME −0.11, *P* < .05) and psychological needs met (AME −0.16, *P* < .01). Educational attainment was positively associated with having spiritual needs met, with both secondary (AME 0.13, *P* < .05) and tertiary education (AME 0.21, *P* < .01) showing higher probabilities compared with primary education.

## Discussion

Our findings reinforce the central role of informal networks in palliative and end-of-life care support. Family members were the main providers across all three domains, and when support was received it was generally perceived as high quality and helpful for coping with illness and approaching death. This aligns with evidence showing that family and friends provide the majority of care for older adults in their final year of life, often delivering sustained practical and emotional support with limited formal input [18, 25]. This reliance on informal caregiving raises concerns about inequities in end-of-life support, as individuals without strong informal networks are at greater risk of unmet non-medical needs.

These findings also raise important questions about who is best placed to provide support for social, psychological, and spiritual needs at the end of life. The high levels of perceived quality and helpfulness reported in our study suggest that informal networks are not only the main providers of such support but may also be particularly well suited to meeting needs that are relational, emotionally grounded, and, in the case of spiritual care, deeply personal. This is especially relevant given that spiritual and existential concerns are often shaped by individual values, life experiences, and social relationships, which may be more readily understood and addressed within close personal networks. Rather than implying that formal health and social care services should assume primary responsibility for these domains, our findings point to the importance of recognising and supporting the role of informal caregivers. The role of formal services may therefore be better conceptualised as complementing, enabling, and coordinating existing support networks, particularly for individuals who lack strong informal ties or whose needs exceed what informal networks can provide.

The strong associations observed for living alone highlight social isolation as a key vulnerability at the end of life. Older adults who live alone may have fewer available caregivers, weaker social ties, or reduced support within care systems, increasing their risk of unmet needs. Previous research has shown that social isolation and living alone are becoming more common among ageing populations and are associated with poorer health outcomes and reduced access to support [26–28]. Our findings suggest that these trends may translate into unequal access to social, psychological, and spiritual support at the end of life, even in a context where clinical care may be available. Individuals with dementia or severe cognitive impairment were also less likely to have social and psychological needs met. This finding is consistent with evidence that people with dementia frequently experience unmet psychosocial needs [29]. Cognitive impairment can limit individuals’ ability to articulate needs, reduce recognition of distress by caregivers, and complicate coordination between services, placing greater strain on informal caregivers and increasing the risk that non-medical needs remain unaddressed [30].

Higher educational attainment was associated with a greater likelihood of having spiritual needs met. This pattern may reflect differences in cultural capital, communication with caregivers, or the ability to articulate and legitimise spiritual concerns within care contexts. Educational gradients in end-of-life experiences have been documented previously, particularly for care preferences and engagement with advance care planning, and may extend to less tangible domains such as spiritual support [31]. Notably, household wealth was not associated with having social, psychological, and spiritual needs met, suggesting that inequalities in this domain do not follow the more commonly observed income gradient in palliative and end-of-life care. This finding underscores that inequalities in end-of-life care are not limited to clinical interventions or place of death but also shape access to non-medical forms of support that contribute to dignity and wellbeing.

The findings have several implications for policy and practice in ageing societies and support recommendations from The Lancet Commission on the Value of Death [17]. First, end-of-life care depends on informal networks. Public health approaches to palliative and end-of-life care recognise this and offer frameworks to build and strengthen informal support [15, 32]. These approaches, including compassionate communities models, emphasise that social, psychological, and spiritual care are shared societal responsibilities rather than the sole responsibility of formal health and social care services [15]. In this perspective, informal networks are not only central providers of support but also key sources of relational and meaningful care, particularly for needs that are deeply personal or socially embedded. At the same time, the role of formal services is to enable, recognise, and work in partnership with these networks, rather than replace them. Second, it is essential to foster closer integration between formal services and informal and community networks. These networks often operate independently [33], but greater coordination and shared responsibility could reduce unmet need, enhance continuity and quality of care, and lead to more sustainable models of palliative and end-of-life care. This is particularly important given existing workforce pressures, as expecting health and social care professionals to take on greater responsibility for holistic care may be difficult to realise without adequate resources. Supporting staff through training, organisational models that allow time for relational care, and attention to wellbeing and burnout is therefore essential to ensure that holistic care is delivered in a meaningful way rather than reduced to a procedural or ‘tick-box’ exercise. Third, inequities exist in availability of, access to, and composition of social networks. Palliative and end-of-life care systems must recognise the influence of social and structural determinants of health in outcomes at the end of life. A routine assessment of living arrangements, social connection, and cognitive status in primary care, community health services, and social care settings could help to identify individuals who may require additional non-medical support as they approach the end of life. Building links with community assets and voluntary organisations can help to connect individuals to networks of support and address unmet needs.

**Table 2:** Sociodemographic and end-of-life factors associated with having social, psychological, and spiritual needs met in the last 3 months of life, ELSA end-of-life interviews.

	Social needs met	Emotional needs met	Spiritual needs met
**Age at death** (55–74)			
75–84	−0.07	0.01	0.00
	(0.05)	(0.06)	(0.09)
85+	−0.04	0.11	0.02
	(0.05)	(0.06)	(0.09)
**Sex** (Male)			
Female	0.05	0.04	−0.07
	(0.04)	(0.04)	(0.06)
**Living alone** (No)			
Yes	−0.08^a^	−0.14^b^	−0.15^a^
	(0.04)	(0.04)	(0.06)
**Education** (Primary)			
Secondary	0.04	0.04	0.13^a^
	(0.04)	(0.04)	(0.06)
Tertiary	0.08	0.05	0.21^b^
	(0.05)	(0.06)	(0.08)
**Standardised non-pension wealth**	−0.02	−0.01	−0.03
	(0.02)	(0.02)	(0.02)
**Place of death** (Home)			
Hospital	−0.04	−0.08	−0.12
	(0.04)	(0.04)	(0.06)
Other	−0.04	−0.05	−0.07
	(0.05)	(0.05)	(0.07)
**Cause of death** (Cancer)			
Cardiovascular related illness	−0.07	−0.07	0.07
	(0.05)	(0.06)	(0.08)
Respiratory disease	−0.02	0.01	0.01
	(0.06)	(0.06)	(0.09)
Other	−0.02	−0.01	0.01
	(0.05)	(0.05)	(0.08)
**Dementia or severe cognitive condition** (No)			
Yes	−0.11^a^	−0.16^b^	−0.15
	(0.05)	(0.06)	(0.08)
Observations	505	483	227

The table shows average marginal effects and standard errors in parentheses. Statistical significance: ^a^*P* < .05, ^b^*P* < .01. The three columns present probit regressions models regressing having social, psychological, and spiritual needs met in the last 3 months of life on sociodemographic and end-of-life variables.

Finally, population ageing, rising rates of dementia, and increasing numbers of older adults living alone suggest that reliance on family care alone may become increasingly unsustainable. Policymakers should anticipate this demographic shift by developing integrated care models that combine medical, social, and community-based responses to ensure equitable end-of-life support across socioeconomic and cognitive vulnerability groups. Without such action, inequalities in non-medical end-of-life care are likely to widen in coming decades.

The study has several limitations. End-of-life interviews rely on proxy respondents, whose reports may not fully capture the deceased individual’s subjective experiences. Proxy respondents’ own emotional state, relationship to the deceased, and involvement in caregiving may influence their perceptions of unmet needs and support quality. In this context, proxy reports may reflect respondents’ own interpretations rather than the deceased individual’s experiences, particularly for psychological and spiritual domains that are inherently subjective. This may influence both the reporting of whether needs were met and the perceived quality of support, potentially leading to over- or under-estimation. These considerations are important when interpreting the findings, as they may shape the patterns observed and the conclusions drawn regarding the adequacy of end-of-life support. In addition, variation in the time elapsed between death and the proxy interview may have introduced recall bias, as respondents were asked to report on experiences occurring in the final months of life after a potentially extended period. Measures of needs being ‘met’ were binary and did not capture gradations in unmet need or differences in the complexity of support required. While analyses adjusted for key sociodemographic and end-of-life characteristics, residual confounding may persist. Finally, findings reflect the English health and social care context and may not generalise to settings with different palliative care infrastructures or cultural norms surrounding end-of-life support.

## Conclusion

Informal networks are the main providers of psychological, social, and spiritual support at the end of life for older adults. Access to these networks is not evenly distributed, creating inequities in end-of-life care outcomes. Older adults who live alone or have dementia are at particular risk of unmet social, psychological, and spiritual needs, underscoring the need to address social and cognitive vulnerability alongside medical care. As populations age and reliance on informal caregiving intensifies, a public health approach that integrates medical, social, and community-based support is essential to ensure that end-of-life care is equitable, sustainable, and responsive to the full range of needs of all individuals approaching death.

## Data Availability

ELSA data are available to registered researchers through the UK Data Service under standard access procedures. No additional data are available from the authors.
